# Public attitudes to implementing financial incentives in stopsmoking services in Ireland

**DOI:** 10.18332/tpc/162364

**Published:** 2023-04-03

**Authors:** Ellen Cosgrave, Aishling Sheridan, Edward Murphy, Martina Blake, Rikke Siersbaek, Sarah Parker, Sara Burke, Frank Doyle, Paul Kavanagh

**Affiliations:** 1 HSE Tobacco Free Ireland Programme, Strategy and Research, HSE, Ireland; 2 Centre for Health Policy and Management, School of Medicine, Trinity College Dublin, The University of Dublin, Dublin 2, Ireland; 3 Department of Health Psychology, School of Population Health, Royal College of Surgeons in Ireland, Dublin 2, Ireland; 4 Department of Public Health and Epidemiology, School of Population Health, Royal College of Surgeons in Ireland, Dublin 2, Ireland

**Keywords:** smoking cessation, motivation, reward, public opinion, MeSH, Ireland

## Abstract

**INTRODUCTION:**

Financial incentives improve stop-smoking service outcomes. Views on acceptability can influence implementation success. To inform implementation planning in Ireland, public attitudes on financial incentives to stop smoking were measured.

**METHODS:**

A cross-sectional telephone survey was administered to 1000 people in Ireland aged ≥15 years in 2022, sampled through random digit dialing. The questionnaire included items on support for financial incentives under different conditions. Prevalence of support was calculated with 95% Confidence Intervals (CIs) and multiple logistic regression identified associated factors using adjusted odds ratios (AORs) with 95% CIs.

**RESULTS:**

Almost half (47.0%, 95% CI: 43.9–50.1) of the participants supported at least one type of financial incentive to stop smoking, with support more prevalent for shopping vouchers (43.3%, 95% CI: 40.3–46.5) than cash payments (32.1%, 95% CI: 29.2–35.0). Support was similar for universal and income-restricted schemes. Of those who supported financial incentives, the majority (60.6%) believed the maximum amount given on proof of stopping smoking should be under €250 (median=100, range: 1–7000). Compared to their counterparts, those of lower education level (AOR=1.49; 95% CI: 1.10–2.03, p=0.010) and tobacco/e-cigarette users (AOR=1.43; 95% CI: 1.02–2.03, p=0.041) were significantly more likely to support either financial incentive type, as were younger people.

**CONCLUSIONS:**

While views on financial incentives to stop smoking in Ireland were mixed, the intervention is more acceptable in groups experiencing the heaviest burden of smoking-related harm and most capacity to benefit. Engagement and communication must be integral to planning for successful implementation to improve stop-smoking service outcomes.

## INTRODUCTION

Smoking continues to cause harm on a huge scale and helping people quit remains a key public health priority^1^. The components of effective stop-smoking support are well-established^2,3^. The challenge for tackling the harms caused by smoking is effective implementation of what works, especially for lower income groups with the greatest burden of smoking-related harm, for whom tailored stop-smoking services have potential pro-equity impact^4^. While financial incentives to stop smoking (FISS) can improve service outcomes^5^, knowledge to guide effective implementation design is lacking^6^.

Planning for implementation success can help translate FISS evidence into better stop-smoking services^7^. Acceptability has been defined as ‘the extent to which people delivering or receiving a healthcare intervention consider it to be appropriate, based on anticipated or experienced cognitive and emotional responses to the intervention across implementation stakeholders’^8^. It is an important facet of implementation success since it can positively influence scalable and sustainable implementation of healthcare interventions^9^. FISS is a controversial approach and can evoke mixed public reactions, with concerns including gaming, manipulation and fairness^10^. Views across the public generally are important where, as in Ireland, stop-smoking services are publicly funded, since low public acceptability can undermine funding decisions by policy-makers and support by healthcare professionals^11^.

Ireland faces challenges with widening social inequalities in smoking^12^. Recently published National Stop Smoking Guidelines identified FISS as a promising intervention^13^, especially for people in lower income groups, but recommended further local research for effective implementation planning. This study aimed to measure perceived acceptability of financial incentives among the Irish public.

## METHODS

A cross-sectional survey of a nationally chosen random sample of 1000 members of the Irish public aged ≥15 years was conducted through a market research company (IPSOS MRBI) in February 2022. Sample size was calculated based on the assumption that 50% of the public would support FISS; a sample size of 784 was sufficient to measure this proportion with a 95% CI of ±3.5%. In total, 3386 people were contacted via random digit-dialing to yield 1000 participants. Participants without a telephone, who were not fluent in the English language and who did not respond to the survey completely were excluded.

A literature-informed instrument measured agreement with statements on FISS in different forms and settings^10,14-16^. Responses were grouped as ‘support’ (‘strongly agree’/‘somewhat agree’), ‘indifferent’ (‘neither agree nor disagree’/‘don't know’) and ‘oppose’ (‘somewhat disagree’/ ‘strongly disagree’). Participants identified a maximum acceptable incentive value. Tobacco or e-cigarette use status and sociodemographic characteristics were also collected. The questions were embedded in a wider questionnaire, surveying public attitudes to the tobacco endgame, which was conducted in line with the methods described above^17^.

Prevalence of key measures were calculated with 95% CIs, which were used to compare responses together with chi-squared testing. Multiple logistic regression identified factors independently associated with FISS support using adjusted odds ratios (AORs) with 95% CIs. Sociodemographic characteristics and tobacco/e-cigarette user status were examined for association with support, using chi-squared testing. Those factors with significance entered the final multiple logistic regression model as categorical variables (age, education level, region, social grade and tobacco/e-cigarette user status) and non-significant variables were retained where these improved model fit (gender) (see data dictionary in the Supplementary file). Re-weighting in line with recent population estimates for gender, age, region and social grade was employed prior to all analyses. Analyses were conducted in IBM SPSS Statistics for Windows Version 26.0.

## RESULTS

Almost half of the participants (47.0%; 95% CI: 43.9–50.1) supported at least one type of FISS, either shopping vouchers or cash payments. Support for shopping vouchers was higher than for cash payments (43.3%; 95% CI: 40.3–46.5 vs 32.1%; 95% CI: 29.2–35.0, χ^2^=27.16, p<0.001). Approximately 1 in 10 was indifferent to cash incentives (9.8%, 95% CI: 8.0–11.6) and to voucher incentives (10.4%, 95% CI: 8.5–12.3) (Supplementary file Table 1).

A similar proportion of participants supported FISS for people who smoke, regardless of their income (unrestricted or universal FISS) and for those on lower incomes (restricted or targeting FISS by social group) (33.0%; 95% CI: 29.1–37.0 vs 32.1%; 95% CI: 28.2–36.1, χ^2^=0.012, p=0.93).

The majority of participants who supported FISS (60.5%; 95% CI: 55.4–65.4) identified a maximum acceptable value under €250 (median=100, range: 1–7000).

Participant age, gender, region of residence, social grade, education level, and tobacco/e-cigarette use status were included in the final multiple logistic regression model to identify factors independently associated with FISS support. Compared to their counterparts, those of lower education level (i.e. less than third level education completed, AOR=1.49; 95% CI: 1.10–2.03) and tobacco/e-cigarette users (AOR=1.43; 95% CI: 1.02–2.03) were significantly more likely to support FISS (Table 1). Participants aged ≥35 years were less likely to support FISS than their younger counterparts, however, there was no association between FISS support and gender.

**Table 1:** Cross-sectional survey of public attitudes to financial incentives to stop smoking, Ireland 2022. Multiple logistic regression analysis of factors associated with participant support for financial incentives (either cash or shopping voucher incentives) (N=1000)

*Characteristics*	*Total n (%)*	Supported financial incentives* n (%)	*Did not support financial incentives n (%)*	*p*	*OR (95% CI)*	*AOR (95% CI)*	*p*
**Gender**							
Female (Ref.)	509 (50.9)	230 (45.2)	279 (54.8)	0.242^a^	1	1	
Male	491 (49.1)	240 (48.9)	251 (51.1)		1.16 (0.91–1.49)	1.07 (0.82–1.39)	0.623
**Age** (years)							
15–24	159 (15.9)	108 (67.9)	51 (32.1)	<0.001^a^	**1.76 (1.11–2.79)**	1.31 (0.80–2.15)	0.289
25–34 (Ref.)	153 (15.3)	84 (54.9)	69 (45.1)		1	1	
35–44	194 (19.4)	82 (42.3)	112 (57.7)		**0.60 (0.39–0.92)**	**0.61 (0.39–0.94)**	**0.026**
45–54	171 (17.1)	60 (35.1)	111 (64.9)		**0.45 (0.29–0.70)**	**0.44 (0.28–0.70)**	**<0.001**
55–64	140 (14.0)	52 (37.1)	88 (62.9)		**0.49 (0.31–0.78)**	**0.43 (0.26–0.70)**	**0.001**
≥65	183 (18.3)	83 (45.4)	100 (54.6)		0.68 (0.44–1.04)	**0.57 (0.36–0.92)**	**0.020**
**Region**							
Leinster (Ref.)	558 (55.8)	266 (47.7)	292 (52.3)	0.040^a^	1	1	
Munster	267 (26.7)	111 (41.6)	156 (58.4)		0.78 (0.58–1.05)	0.82 (0.61–1.12)	0.214
Connaught/Ulster	175 (17.5)	94 (53.7)	81 (46.3)		1.28 (0.91–1.80)	1.31 (0.92–1.88)	0.140
Social grade*							
Higher (A, B, C1) (Ref.)	435 (43.5)	183 (42.1)	252 (57.9)	0.011^a^	1	1	
Lower (C2, D, E)	505 (50.5)	261 (51.7)	244 (48.3)		1.43 (0.83–2.46)	1.21 (0.68–2.13)	0.516
Farmer	60 (6.0)	26 (43.3)	34 (56.7)		0.98 (0.57–1.69)	1.00 (0.56–1.79)	0.995
Education level*							
Higher (Ref.)	544 (54.4)	218 (40.1)	326 (55.9)	<0.001^a^	1	1	
Lower	456 (45.6)	252 (55.3)	204 (44.7)		**1.85 (1.44–2.38)**	**1.49 (1.10–2.03)**	**0.010**
**Tobacco/e-cigarette user**							
No (Ref.)	802 (80.2)	358 (44.6)	444 (55.4)	0.002^a^	1	1	
Yes	192 (19.2)	110 (57.3)	82 (42.7)		**1.67 (1.22–2.30)**	**1.43 (1.02–2.03)**	**0.041**

Analyses based on weighted data. AOR: adjusted odds ratio; factors included in the fully adjusted model were gender, age, region, social grade, education level, and tobacco/e-cigarette user status.

* A data dictionary is provided in the Supplementary file.

a Chi-squared test.

Bold: p<0.05. Naglekerke r^2^= 0.095.

## DISCUSSION

FISS is a promising approach to improving stop-smoking service outcomes^5^. Our study assessed public attitudes for the first time in Ireland to inform implementation planning. Views on FISS acceptability were mixed; however, the intervention was more acceptable in groups experiencing the greatest burden of smoking-related harm who have most capacity to benefit. There was higher support for shopping vouchers than cash payments. Support for targeting FISS to people of low income was similar to support for a universal approach. Potential FISS value that would be supported has been delineated in Ireland, with values of less than €250 being most popular.

Public opinion on FISS has been surveyed in a number of countries to support implementation planning^10,14-16^. A recent systematic review found that views on acceptability of financial incentives for health-related behavior change can be polarised^10^. Public views in Ireland align with these studies. Our findings that vouchers were more acceptable than cash, and that lower maximum incentives values are preferred, are also consistent with reviewed studies^10^.

Concerns regarding fairness commonly arise in literature on financial incentives^10^. In Ireland, as in many high-income countries, the social patterning of smoking is increasing and leading to widening of social inequalities in health^11^. Using FISS to target and tailor stop-smoking services for lower income groups has potential pro-equity impact^4^, and is a critical implementation design decision point. In this study, support for universal FISS and for targeting to people of lower income was similar; in other studies, universal approaches were often more acceptable to the general public^10^. However, we also found that groups in Ireland with greatest need and most capacity to benefit from FISS (younger people with lower education level who smoke) were more likely to report support. In other studies, acceptability was not always higher among groups with more capacity to benefit from financial incentives to help change unhealthy behaviours^16,18^. Compared to universal approaches, pursuing equity through targeting FISS to people with lower income may lead to friction or trade-offs in acceptability across stakeholders groups^10^. While this approach may evoke mixed reactions across the public generally, many of whom may not need the service, targeting FISS to those with lower income who smoke may be more acceptable in this group who urgently need improved stop-smoking services to address widening health inequalities.

Poor public acceptability of FISS can threaten implementation success^11^. Even people who smoke report concerns about potential abuse of FISS and fairness^14^. Stop-smoking services in Ireland are publicly funded, meaning the public generally are key stakeholders in implementation planning. Participants were not provided with information on intervention rationale or arguments for targeting FISS for lower income groups. These messages matter. For example, a discrete choice experiment found FISS acceptability increased when information effectiveness was provided^19^.

This is the first study in Ireland to measure acceptability of financial incentives in stop-smoking services. International evidence is useful, but contextually relevant research is needed to inform stakeholder communication and engagement for local implementation success, since social context influences views on financial incentives acceptability^15^, and media representation also shapes opinions of the intervention^20^.

### Limitations

The study has key limitations. The response rate was low, potentially impacting generalizability. Information on FISS effectiveness and rationale, which affect acceptability, was not presented. Finally, the narrow question set did not explore reasons for respondent views. This research will benefit from complementary qualitative studies to provide richer evidence on this complex challenge, which are planned.

## CONCLUSIONS

Translating research evidence on FISS into better outcomes for those with greatest need is a complex challenge. Understanding acceptability can help plan for implementation success. While views of the Irish public on FISS were mixed, acceptability was greater among groups who will benefit most from the improvement in stop-smoking service effectiveness. These findings highlight a need for stakeholder engagement and communication in FISS implementation planning. Co-design of FISS scheme (including final decision on value, staging over time, and verification processes) is planned in Ireland and will be followed by careful FISS piloting to demonstrate effectiveness and address concerns to build support and sustain successful implementation prior to more widespread scaling.

## Data Availability

The data supporting this research are available from the authors on reasonable request.
