# Supplementary material for: Public attitudes to implementing financial incentives in stopsmoking services in Ireland
Source: Tob Prev Cessat. 2023 Apr 3;9:09. doi: 10.18332/tpc/162364 (PMC10068872; doi:10.18332/tpc/162364)
Supplement: Supplementary file 1 [file TPC-9-09-s1.pdf]

SUPPLEMENTARY MATERIALS

Table: Cross-Sectional Survey of Public Attitudes to Financial Incentives to Stop Smoking, Ireland 2022 (n=1,000). Support for Financial Incentives According to Tobacco/E-cigarette Use Status (N=1,000)

Measure	Total n (%)	*Tobacco/e-cigarette User n (%)*	*Non-Tobacco/e-cigarette User n (%)*
**"Shopping vouchers should be provided to people who prove that they have stopped smoking"**			
Agree	434 (43.4)	*96 (49.7)*	*337 (42.0)*
Indifferent	98 (9.8)	*20 (10.4)*	*78 (9.7)*
Disagree	469 (46.9)	*77 (39.9)*	*388 (48.3)*
**"A cash payment should be provided to people who prove that they have stopped smoking"**			
Agree	321 (32.1)	*79 (40.9)*	*242 (30.2)*
Indifferent	104 (10.4)	*22 (11.4)*	*82 (10.2)*
Disagree	575 (57.5)	*92 (47.7)*	*477 (59.6)*

Note: Due to the application of weights as described in methods, the total column for "shopping vouchers" totals 1,001.

Table: Cross-Sectional Survey of Public Attitudes to Financial Incentives to Stop Smoking, Ireland 2022 (n=1,000). Questionnaire content.

Question	Responses
**Q.1. Could I ask what age group you fall into?**	1=15-17, 2=18-24, 3=25-34, 4=35-44, 5=45-54, 6=55-64, 7=65+
**Q.2. Are you male or female?**	1=Male, 2=Female
**Q.3. What region are you living in?**	1=Dublin, 2=Rest of Leinster, 3=Munster, 4=Connaught/Ulster
**Q.4. To ensure we interview a wide cross-section of the public, could I first ask what the occupation of the chief income earner in your home is?**	1=AB, 2=C1, 3=C2, 4=DE, 5=F
**Q.5. What is the highest level of education you have completed to date?**	1=Completed primary school, 2=Currently in secondary school, 3=Completed secondary school, 4=Currently at third level, 5=Completed third level, 6=No formal education
**Q.6. Do you smoke tobacco products? For the purposes of this survey, tobacco products do not include e-cigarettes.**	1=Yes daily smoker, 2=Yes occasionally, 3=No, 4=Don't know
**Q.7. Which of the following statements BEST applies to you?**	1=I have never tried e-cigarettes, 2=I have tried e-cigarettes but do not use them anymore, 3=I have tried e-cigarettes and use them daily, 4=I have tried e-cigarettes and use them occasionally, 5=Don't know
**Q.8. Now I am going to put forward two potential incentives that could be used to encourage people to stop smoking. To what extent do you agree or disagree with the following satement: Shopping vouchers should be provided to people who prove that they have stopped smoking.**	1=Strongly agree, 2=Somewhat agree, 3=Neither agree nor disagree, 4=Somewhat disagree, 5=Strongly disagree, 6=Don't know
**Q.9. Now I am going to put forward two potential incentives that could be used to encourage people to stop smoking. To what extent do you agree or disagree with the following satement: A cash payment should be provided to people who prove that they have stopped smoking**	1=Strongly agree, 2=Somewhat agree, 3=Neither agree nor disagree, 4=Somewhat disagree, 5=Strongly disagree, 6=Don't know
**Q.10. What is the maximum amount you feel should be given as an incentive to people who prove that they have stopped smoking?**	INTERVIEWER RECORDS AMOUNT IN EURO<br><br>€_________________

Note: Further information on social grading used by the market research provider is available here: https://www.ipsos.com/sites/default/files/publication/6800-03/MediaCT_thoughtpiece_Social_Grade_July09_V3_WEB.pdf

Table: Cross-Sectional Survey of Public Attitudes to Financial Incentives to Stop Smoking, Ireland 2022 (n=1,000). Further details on social grades.

**Social Grade Group A**	Professional people, very senior managers in business or commerce or top-level civil servants. Retired people and their widows, previously grade A.
**Social Grade Group B**	Middle management executives in large organisations with appropriate qualifications. Principal officers in local government and the civil service. Top management or owners of small business, education and service establishments. Retired people, and their widows, previously grade B.
**Social Grade Group C1**	Junior management, owners of small establishments, and all others in non-manual positions. Retired people, and their widows, previously grade C1.
**Social Grade Group C2**	All skilled manual workers, and those manual workers with responsibility for other people. Retired people, and their widows, previously grade C2, with pensions from their job/late husband's job.
**Social Grade Group D**	All semi-skilled and unskilled manual workers, and apprentices and trainees to skilled workers. Retired people, and their widows, previously grade D, with pensions from their job/late husband's job.
**Social Grade Group E**	All those entirely dependent on the state long-term, through sickness, unemployment, old age or other reasons. Retired persons who receive only the standard basic state pension. Widows who receive only widows benefit. Those unemployed for a period exceeding six months. Casual or intermittent workers and those without a regular income.
**Social Grade Group F**	F1 - Farmers or farm managers of holdings of 50 acres or more and their dependants.<br>F2 - Farmers or farm managers of holdings of less than 50 acres. Farm workers and farm labourers and their dependants.

Table: Cross-Sectional Survey of Public Attitudes to Financial Incentives to Stop Smoking, Ireland 2022 (n=1,000). Data dictionary.

No.	Variable	Type of variable	Description	Original Coding	Recoding
**1.**	Age (years)	Categorical	Age group of participant	1=15-17, 2=18-24, 3=25-34, 4=35-44, 5=45-54, 6=55-64, 7=65+	1=15-24 (1,2), 2=25-44 (3,4), 3=45-64 (5,6), 4=65+ (7)
**2.**	Gender	Categorical	Gender of participant	1=Male, 2=Female	0=Female (2), 1=Male (1)
**3.**	Region	Categorical	Region where participant resides	1=Dublin, 2=Rest of Leinster, 3=Munster, 4=Connaught/Ulster	0=Leinster (1,2), 1=Munster (3), 2=Connaught/Ulster (4)
**4.**	Social class	Categorical	Social class of participant	1=AB, 2=C1, 3=C2, 4=DE, 5=F	0=Higher (1,2), 1=Lower (3,4), 2=Farmer (5)
**5.**	Educational attainment	Categorical	Highest level of education attained	1=Completed primary school, 2=Currently in secondary school, 3=Completed secondary school, 4=Currently at third level, 5=Completed third level, 6=No formal education	0=Higher (5), 1=Lower (1-4,6)
**6.**	Current smoking status	Categorical	Whether participant currently smokes cigarettes or tobacco products	1=Yes daily smoker, 2=Yes occasionally, 3=No, 4=Don’t know	0=Non-smoker (3), 1=Smoker (1,2), Missing=Don’t know (4)
**7.**	Current E-cigarette use status	Categorical	Whether participant currently uses e-cigarettes	1=I have never tried e-cigarettes, 2=I have tried e-cigarettes but do not use them anymore, 3=I have tried e-cigarettes and use them daily, 4=I have tried e-cigarettes and use them occasionally, 5=Don’t know	0=Non-e-cigarette user (1,2), 1=E-cigarette user (3,4), Missing=Don’t know (5)
**8.**	Current tobacco/e-cigarette use status	Categorical	Whether participant currently uses tobacco products or e-cigarettes	Composite variable derived from variables 6. And 7.	0=Non-tobacco/e-cigarette user, 1=Tobacco/e-cigarette user, Missing=Don’t know
**9.**	TFI goal support	Categorical	Whether participant supports the TFI goal	1=Strongly agree, 2=Somewhat agree, 3=Neither agree nor disagree, 4=Somewhat disagree, 5=Strongly disagree, 6=Don’t know	0=No support (3,4,5,6), 1=Support (1,2)

No.	Variable	Type of variable	Description	Original Coding	Recoding
**10.**	Support for a tobacco sales phase-out	Categorical	Whether participants supported a tobacco sales phase-out	1=Support with no conditions, 2=Support if the government provides assistance to help smokers to quit, 3=Support but only if existing smokers can continue to buy tobacco products using a licence, 4=Support but only if conditions in both 2. and 3. are met , 5=Does not support , 6=Don't know	0=No support(5,6), 1=Support (1,2,3,4)
**11.**	Support for shopping voucher incentives	Categorical	Whether shopping vouchers should be provided to people who prove that they have stopped smoking	1=Strongly agree, 2=Somewhat agree, 3=Neither agree nor disagree, 4=Somewhat disagree, 5=Strongly disagree, 6=Don't know	0=No support (3,4,5,6), 1=Support (1,2)
**12.**	Support for cash incentives	Categorical	Whether A cash payment should be provided to people who prove that they have stopped smoking	1=Strongly agree, 2=Somewhat agree, 3=Neither agree nor disagree, 4=Somewhat disagree, 5=Strongly disagree, 6=Don't know	0=No support (3,4,5,6), 1=Support (1,2)
**13.**	Maximum incentive amount acceptable	Numerical	The maximum amount partipipants feel should be given as an incentive to people who prove that they have stopped smoking	Enter as given	1= <50 euro<br>2=50-99 euro<br>3=100-149 euro<br>4=150-199 euro<br>5=200-249 euro<br>6=250-299 euro<br>7=300-349 euro<br>8=350-399 euro<br>9=400-449 euro<br>10=500-999 euro<br>11=>/=1000 euro

© 2023 Cosgrave E. et al.
